# Attenuation of the Severity of Acute Respiratory Distress Syndrome by Pomiferin through Blocking Inflammation and Oxidative Stress in an AKT/Foxo1 Pathway-Dependent Manner

**DOI:** 10.1155/2022/5236908

**Published:** 2022-11-26

**Authors:** Zheng Tang, Zetian Yang, Hui Feng, Xuefeng Zhou, Ming Mao

**Affiliations:** ^1^Department of Thoracic Surgery, Zhongnan Hospital of Wuhan University, Wuhan, Hubei, China; ^2^Information Center, Renmin Hospital of Wuhan University, Wuhan, Hubei, China

## Abstract

Acute respiratory distress syndrome (ARDS) gives rise to uncontrolled inflammatory response and oxidative stress, causing very high mortality globally. Pomiferin is a kind of prenylated isoflavonoid extracted from *Maclura pomifera*, owning anti-inflammatory and antioxidant properties. However, the functions and possible mechanisms of pomiferin in lipopolysaccharide- (LPS-) induced ARDS remain unknown. C57BL/6 mice were injected with LPS (5 mg/kg) intratracheally to induce an *in vivo* ARDS model while RAW264.7 macrophages were stimulated with LPS (100 ng/ml) to induce an *in vitro* model. Our data demonstrated that pomiferin (20 mg/kg) significantly improved pulmonary function and lung pathological injury in mice with ARDS, apart from increasing survival rate. Meanwhile, pomiferin treatment also inhibited LPS-induced inflammation as well as oxidative stress in lung tissues. LPS stimulation significantly activated AKT/Foxo1 signal pathway in lung tissues, which could be reversed after pomiferin treatment. *In vitro* experiments further showed that 10, 20, and 50 *μ*M of pomiferin could enhance cell viability of RAW264.7 macrophages stimulated with LPS. What is more, 3-deoxysappanchalcone (3-DE), one AKT agonist, was used to active AKT in RAW264.7 macrophages. The results further showed that 3-DE could abolish pomiferin-elicited protection in LPS-treated RAW264.7 macrophages, evidenced by activated inflammation and oxidative stress. Taken together, our study showed that pomiferin could exert an ARDS-protective effect by blocking the AKT/Foxo1 signal pathway to inhibit LPS-induced inflammatory response and oxidative injury, which may serve as a potential candidate for the treatment of ARDS in the future.

## 1. Introduction

Acute respiratory distress syndrome (ARDS) is a dangerous and life-threatening pathological condition which prevents enough oxygen from getting into the lungs and blood, serving as a serious and medical problem urgently needed solving [1]. In addition to bacterial and viral pneumonia, some nonpulmonary sources involving aspiration of gastric contents, severe trauma, drug reaction, and pancreatitis are all related with the development of ARDS [2–4]. The Berlin definition diagnostic criteria for ARDS involve arterial hypoxemia with PaO_2_/FiO_2_ ratio < 300 mmHg and bilateral infiltrates without cardiogenic pulmonary edema on chest imaging [5]. In spite of several decades of investigation on ARDS, there are still no effective drugs and apart from primarily supportive treatment such as a conservative fluid management and lung protective ventilation [6]. Hence, it is significant to explore novel drugs or chemical components which could effectively alleviate the severity of ARDS at present.

There are three pathophysiologic derangements driving the development of ARDS, namely, hyperactivated inflammation, increased lung endothelial permeability, and damaged epithelial barrier [7–9]. In the inception phase, acute lung pathological injury is mainly triggered by dysregulated inflammation. To be more specific, microbial products or danger associated molecular patterns (DAMPs) bind to toll-like receptors (TLRs) on the alveolar macrophages, subsequently activating the innate immunity [10, 11]. Then, the activated immune system further generates reactive oxygen species (ROS) and cytokines which can further give rise to worsening lung injury [12]. AKT/Foxo1 signaling pathway possesses multiple regulators as well as effectors including glucose, insulin, cytokines, and growth factors, displaying significant functions in cell survival, metabolism, inflammation, and oxidative stress [13–16]. Previous studies have demonstrated that changes in AKT activity levels in macrophages show significant impacts on polarization and activation of macrophages via regulating Foxo1 activity during ARDS [17, 18]. Hence, AKT/Foxo1 pathway may be a critical signaling target in drug discovery and treatment of ARDS.

Pomiferin serves as a prenylated isoflavonoid originally extracted from the fruits of *Maclura pomifera* in 1939 by Wolfrom et al. [19]. Till, multiple pharmacological effects of pomiferin have been identified, including anti-inflammation [20], antioxidation [21], and anticancer property [22]. What is more, Bajer and colleagues reported that pomiferin could inhibit insulin-like growth factor-induced activation of AKT [23]. Based on these facts, we speculate that pomiferin may exert pulmonary protection during ARDS by regulating AKT activity. Our study was designed to access the role of pomiferin in the severity of lipopolysaccharide- (LPS-) induced inflammation and oxidative stress during ARDS, aiming to provide evidence-based proofs for clinical application of pomiferin as a novel candidate against ARDS in the future.

## 2. Materials and Methods

### 2.1. Animals and Treatment

Male specific-pathogen-free C57BL/6 mice (6 ~ 8 weeks old, 22.3 ~ 25.6 g) were provided by Institute of Laboratory Animal Science, Chinese Academy of Medical Sciences (Beijing, China). Animal care and experiments were approved by Ethics Committee of the Wuhan University (Wuhan, China) and conducted in accordance with the Guide for the Care and Use of Laboratory Animals of the National Institutes of Health. To establish a murine ARDS model, mice were injected with LPS (5 mg/kg) (Sigma Aldrich, St. Louis, Missouri) intratracheally via a MicroSprayer syringe assembly (MSA-250-M, Penn Century, USA) on the basis of a previous study [24]. One week before intratracheal administration of LPS, mice were administered intragastrically with pomiferin (20 mg/kg/day) for 7 consecutive days. After LPS stimulation for twelve hours, arterial blood gas and pulmonary function were detected. Whereafter, lung tissues were collected from mice under anesthesia for subsequent detection.

### 2.2. Kaplan-Meier Survival Analysis

Another forty mice that had free access to food and water were used to access their survival condition. Mice were divided into four groups, namely, control group, pomiferin group, ARDS group, and ARDS + pomiferin group. Before a lethal dose of LPS stimulation (15 mg/kg), mice were pretreated with pomiferin (20 mg/kg/day) for 7 consecutive days intragastrically. The death number was tracked at the same time point every day. The survival rate carve was drawn after 7 days.

### 2.3. Pulmonary Function and Arterial Blood Gas Analysis

Pulmonary function was recorded using Buxco® Research Systems, Data Sciences International (St. Paul, MN, USA). To be more specific, mice were tracheostomized and inserted with a tracheal cannula containing a computer-controlled ventilator under anesthesia. Subsequently, tidal volume, pulmonary ventilation, and compliance were determined on the basis of previous description [25].

As for arterial blood gas analysis, arterial blood was extracted via left ventricle puncture, followed by immediate arterial blood gas analysis via an automatic blood gas analyzer (ABL80, Denmark). Arterial oxygen partial pressure (PaO_2_), arterial carbon dioxide partial pressure (PaCO_2_), and sodium bicarbonate (HCO_3_^−^) were collected to reflect the status of pulmonary function and acid-base equilibrium.

### 2.4. H&E Staining

The lung tissues from mice were embedded in paraffin, which were then cut into 5 *μ*m slices. These lung tissue sections were stained with hematoxylin and eosin (H&E). Finally, the stained sections were observed under a light microscope (Olympus, Tokyo, Japan). According to the observed pulmonary hemorrhage, inflammatory infiltration, and interstitial edema, lung injury score was evaluated and calculated. On the basis of the severity of the pathologic change, 0 represents no apparent lesions, 0.5–1 represents mild lesions, 2 represents moderate lesions, 3 represents severe lesions, and 4 represents critical lesion. The final score of each sample was calculated by summing up the score of each indicator [26].

### 2.5. Lung Wet/Dry Ratio

The intact right lungs were excised from mice after cervical dislocation under anesthesia. Next, the wet weight of each sample was recorded. Subsequently, the right lungs were dried in an oven (60°C) for 48 h, followed by the detection of dry weight. Finally, lung wet/dry ratio was calculated.

### 2.6. Cell Culture and Treatment

The macrophage cell line RAW264.7 was obtained from ATCC (Manassas, VA, USA), which was cultured in Roswell Park Memorial Institute- (RPMI-) 1640 medium containing 10% fetal bovine serum (FBS). The RAW264.7 macrophages were treated with 100 ng/ml of LPS for 24 h at 37°C to establish cellular ARDS models [27]. To study the potential effects of pomiferin on oxidative stress and inflammatory response in macrophages, pomiferin (50 *μ*M) was used to treat the RAW264.7 macrophages stimulated with LPS. To active AKT, 30 *μ*M of 3-deoxysappanchalcone (3-DE, #HY-N1745A, Shanghai, MCE, China) was used to pretreat RAW264.7 macrophages for 30 min [28].

### 2.7. Cell Viability

RAW264.7 macrophages were seeded into a 96-well plate with 100 *μ*l of culture medium. 10 *μ*l various concentrations (0.1, 10, 20, and 50 *μ*M) of pomiferin was used to incubate macrophages with or without LPS (100 ng/ml) stimulation. Next, CCK8 reagent (#CK04, Dojindo, Japan) was added into each well to incubate the cells for 2 h. Cell viability was reflected by the optical density (OD) value using a microplate reader at the wavelength of 450 nm.

### 2.8. Real-Time Quantitative Polymerase Chain Reaction (RT-qPCR)

Total RNA was extracted from RAW264.7 macrophages and the lung tissues using Trizol reagent (#93289, Sigma-Aldrich), which was then transcribed reversely to cDNA on the basis of standard protocols. After that, the concentration and purity of RNA were detected by 260/280 nm absorbance. Next, SYBR Green PCR kits were applied for RT-qPCR using a deep-well real-time PCR detection system. The relative mRNA levels of genes were normalized with the level of *Gapdh* in the same group. The primers used in this study are presented in Table 1.

### 2.9. Western Blot

Protein samples from RAW264.7 macrophages or lung tissues were washed with PBS, followed by the lysis on ice in lysis buffer containing phosphatase inhibitors and protease inhibitors. Next, the samples were centrifuged (12,000 × g) at 4°C for 15 min. Equal amounts of protein were separated on SDS-PAGE gel, which were then transferred onto polyvinylidene difluoride membranes. After that, 5% nonfat milk was used to block the membranes for 90 min at room temperature, followed by the incubation with primary antibodies at 4°C overnight. Then, the membranes were incubated with peroxidase-conjugated secondary antibodies at room temperature for 60 min. At last, the grey value of these protein bands was scanned and quantified using Image Lab software (BioRad, USA). The primary antibodies as well as their dilution ratio are displayed as follows: SOD1 (#ab183881, Abcam, 1 : 1000), SOD2 (#ab68155, Abcam, 1 : 1000), GPX4 (#ab252833, Abcam, 1 : 500), GAPDH (#ab8245, Abcam, 1 : 1000), phosphorylated-AKT (p-AKT) (#ab38449, Abcam, 1 : 500), AKT (#ab18785, Abcam, 1 : 500), p-Foxo1 (#ab259337, Abcam, 1 : 500), and Foxo1(#ab179450, Abcam, 1 : 500). The relative protein levels of genes were normalized with the level of GAPDH in the same group.

### 2.10. Statistical Analysis

All data are displayed as the means ± standard deviation (SD), which are then analyzed using SPSS 22.0 software (SPSS Inc., USA). Differences among 3 or more groups were compared via one-way ANOVA followed by the Newman–Keuls post hoc test while differences between two groups were analyzed via an unpaired two-sided Student's *t*-test. Differences were regarded statistically significant if *P* < 0.05.

## 3. Results

### 3.1. Pomiferin Treatment Significantly Increased 7-Day Survival Rate of Mice with ARDS

**Figure 1:**
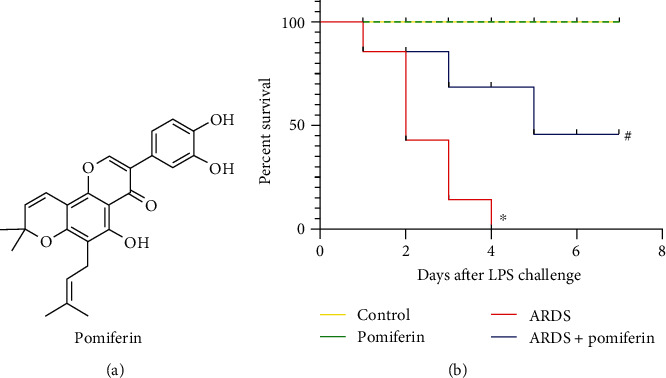
Pomiferin treatment significantly increased 7-day survival rate of mice with ARDS. 7-day survival rate in indicated groups. Before a lethal dose of LPS stimulation (15 mg/kg), mice were pretreated with pomiferin (20 mg/kg/day) for 7 consecutive days intragastrically. The death number was tracked at the same time point every day (*n* = 10). The survival rate carve was drawn after 7 days. ^∗^*P* < 0.05 vs. control group, ^#^*P* < 0.05 vs. ARDS group.

To observe the effect of pomiferin on survival condition of mice with ARDS, a lethal dose of LPS (15 mg/kg) was injected after pomiferin (20 mg/kg/day) was pretreated for 7 consecutive days intragastrically. As shown in Figure 1, LPS stimulation significantly gave rise to a higher mortality. On the fourth day, the mortality reached 100% in ARDS group. However, pomiferin pretreatment significantly improved 7-day survival rate of mice with ARSD, with a 50% survival rate after 7 days.

### 3.2. Pomiferin Treatment Significantly Improved Pulmonary Function and Acid-Base Equilibrium in Mice with ARDS

**Figure 2:**
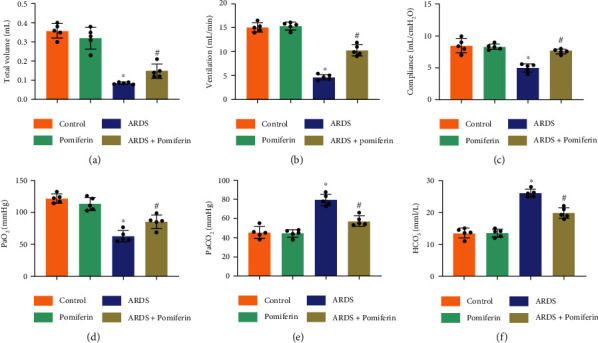
Pomiferin treatment significantly improved pulmonary function and acid-base equilibrium in mice with ARDS. (a–c) Mice were injected with LPS (5 mg/kg) intratracheally treated with or without pomiferin (20 mg/kg/day) intragastrically. Noninvasive pulmonary functional parameters including total volume, ventilation, and lung compliance were detected (*n* = 5). (d, e) Arterial blood gas analysis of PaO_2_, PaCO_2_, and HCO_3_^−^ (*n* = 5). ^∗^*P* < 0.05 vs. control group, ^#^*P* < 0.05 vs. ARDS group.

ARDS is featured by pulmonary dysfunction and acid-base disequilibrium in artery blood [29]. Mice with ARDS had poor pulmonary function, evidenced by decreased tidal volume, ventilation, and pulmonary compliance (Figures 2(a)–2(c)). After pomiferin pretreated for 7 days, tidal volume, ventilation, and pulmonary compliance significantly improved in mice stimulated with LPS. In addition, blood gas analysis showed that LPS stimulation obviously decreased PaO_2_ but increased PaCO_2_ and HCO_3_^−^ in arterial blood from mice. But pomiferin treatment could maintain acid-base equilibrium (Figures 2(d)–2(f)). Taken together, these data indicate that pomiferin treatment significantly improved pulmonary function and acid-base equilibrium in mice with ARDS.

### 3.3. Pomiferin Treatment Significantly Alleviated Lung Pathological Injury in Mice with ARDS

**Figure 3:**
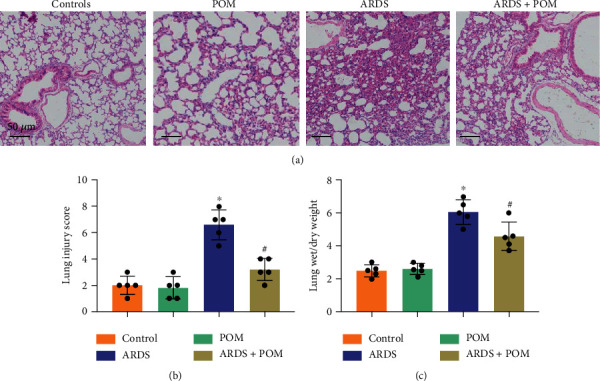
Pomiferin treatment significantly alleviated lung pathological injury in mice with ARDS. (a, b) H&E staining and lung injury score in indicated groups (*n* = 5). (c, d) Lung wet/dry weight in indicated groups (*n* = 5). ^∗^*P* < 0.05 vs. control group, ^#^*P* < 0.05 vs. ARDS group.

Next, we accessed the degree of pathological injury in lung tissues via H&E staining and performed a semiquantitative score on the basis of pulmonary hemorrhage, inflammatory infiltration, and interstitial edema. H&E staining and lung injury score (Figures 3(a) and 3(b)) showed that mice with ARDS showed remarkable inflammation and edema in lung tissues, owning a higher lung injury score than those in control group. Compared with ARDS group, mice in ARDS + pomiferin group showed alleviative lung pathological injury. Also, lung wet/dry weight ratio was also investigated. The results showed that pomiferin treatment could significantly inhibit the increase of lung wet/dry weight ratio in LPS-treated mice (Figure 3(c)). Collectively, the data in this section suggest that pomiferin treatment significantly alleviated lung pathological injury in mice with ARDS.

### 3.4. Pomiferin Treatment Suppressed Inflammation and Oxidative Stress in Lung Tissues from Mice with ARDS

**Figure 4:**
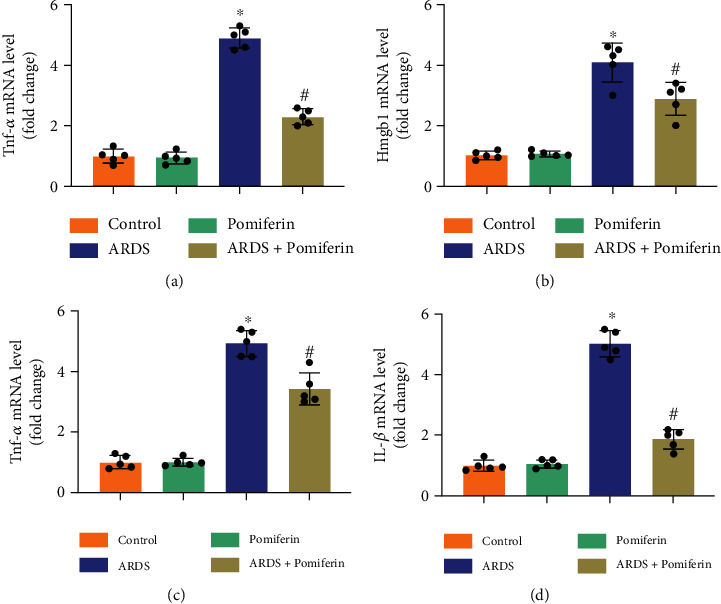
Pomiferin treatment suppressed inflammation in lung tissues from mice with ARDS. (a–d) The mRNA levels of *Tnf-α*, *Hmgb1*, *Mcp-1*, and *Il-1β* in lung tissues determined by RT-qPCR (*n* = 5). ^∗^*P* < 0.05 vs. control group, ^#^*P* < 0.05 vs. ARDS group.

**Figure 5:**
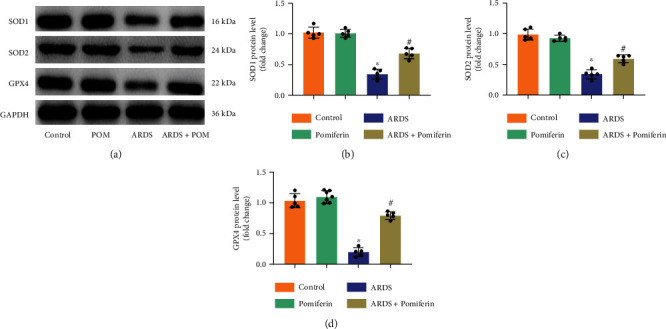
Pomiferin treatment alleviated oxidative stress in lung tissues from mice with ARDS. (a–d) Representative western blots and semi-quantitative results of proteins of SOD1, SOD2, and GPX4 in lung tissues (*n* = 5). ^∗^*P* < 0.05 vs. control group, ^#^*P* < 0.05 vs. ARDS group.

During ARDS, LPS can induce severe oxidative stress by increasing oxidative damage to lipids, proteins, and alveolar-capillary barrier permeability [30]. Therefore, we next detected the mRNA levels of proinflammatory genes and the proteins associated with oxidative stress in lung tissues from indicated groups via RT-qPCR and western blot. The results showed that pomiferin treatment could inhibit the mRNA upregulation of proinflammatory genes, evidenced by decreased mRNA levels of *Tnf-α*, *Hmgb1*, *Mcp-1*, and *Il-1β* (Figures 4(a)–4(d)). Meanwhile, lung tissues from mice with ARDS showed lower levels of SOD1, SOD2, and GPX4 compared with mice from control group, indicating that LPS stimulation inhibited the expression of antioxidant proteins. Compared with ARDS group, the levels of SOD1, SOD2, and GPX4 in lung tissues from ARDS + pomiferin were significantly upregulated (Figures 5(a)–5(d)). These results demonstrated that pomiferin possessed anti-inflammatory and antioxidant potential during ARDS.

### 3.5. Pomiferin Treatment Inhibited the Activation of AKT/Foxo1 Pathway in Lung Tissues from Mice with ARDS

**Figure 6:**
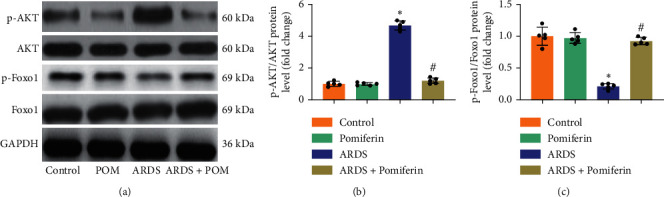
Pomiferin treatment inhibited the activation of AKT/Foxo1 pathway in lung tissues from mice with ARDS. (a–c) Representative western blots and semiquantitative results of proteins of p-AKT, AKT, p-Foxo1, and Foxo1 in lung tissues (*n* = 5). ^∗^*P* < 0.05 vs. control group, ^#^*P* < 0.05 vs. ARDS group.

Previous studies have unveiled that activated AKT could suppress Foxo1 activity, giving rise to a decline in the levels of Foxo1-mediated antioxidant enzymes including catalase and MnSOD, as well as genes associated with proinflammatory mediators [31, 32]. Western blot showed that LPS stimulation could obviously promote the phosphorylation of AKT and decrease the phosphorylation of Foxo1 in lung tissues. As expected, pomiferin treatment blocked the activation of AKT/Foxo1 pathway in lung tissues from mice with ARDS (Figures 6(a)–6(c)). These results demonstrated that AKT/Foxo1 pathway may be involved in the protection mediated by pomiferin during ARDS.

### 3.6. Pomiferin Maintained Cell Viability in LPS-Induced RAW264.7 Macrophages in a Concentration-Dependent Manner

**Figure 7:**
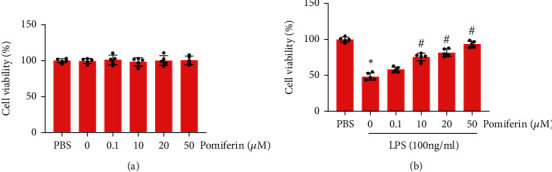
Pomiferin maintained cell viability in LPS-induced RAW264.7 macrophages in a concentration-dependent manner. (a) The effect of different concentrations of pomiferin on cell viability in RAW264.7 macrophages (*n* = 5). (b) The effect of different concentrations of pomiferin on cell viability in LPS-treated RAW264.7 macrophages (*n* = 5). ^∗^*P* < 0.05 vs. PBS group, ^#^*P* < 0.05 vs. pomiferin (0 *μ*M) group.

To confirm the protective role of pomiferin in ARDS, we next observed the effect of pomiferin on cell viability in LPS-treated RAW264.7 macrophages. To begin with, different concentrations of pomiferin were used to stimulate RAW264.7 macrophages for 24 hours. The result (Figure 7(a)) showed pomiferin (0.1, 10, 20, and 50 *μ*M) treatment for 24 hours displayed no effects on cell viability in RAW264.7 macrophages. Additionally, we also observed the effects of pomiferin (0.1, 10, 20, and 50 *μ*M) on cell viability in RAW264.7 macrophages in the context of LPS stimulation. As shown in Figure 7(b), 10, 20, and 50 *μ*M of pomiferin could improve cell viability in LPS-induced RAW264.7 macrophages in a concentration-dependent manner. Thus, 50 *μ*M of pomiferin was selected to treat the RAW264.7 macrophages stimulated with LPS in subsequent *in vitro* experiments.

### 3.7. Pomiferin Inhibited LPS-Induced Inflammation and Oxidative Stress in RAW264.7 Macrophages in an AKT/Foxo1 Pathway-Dependent Manner

**Figure 8:**
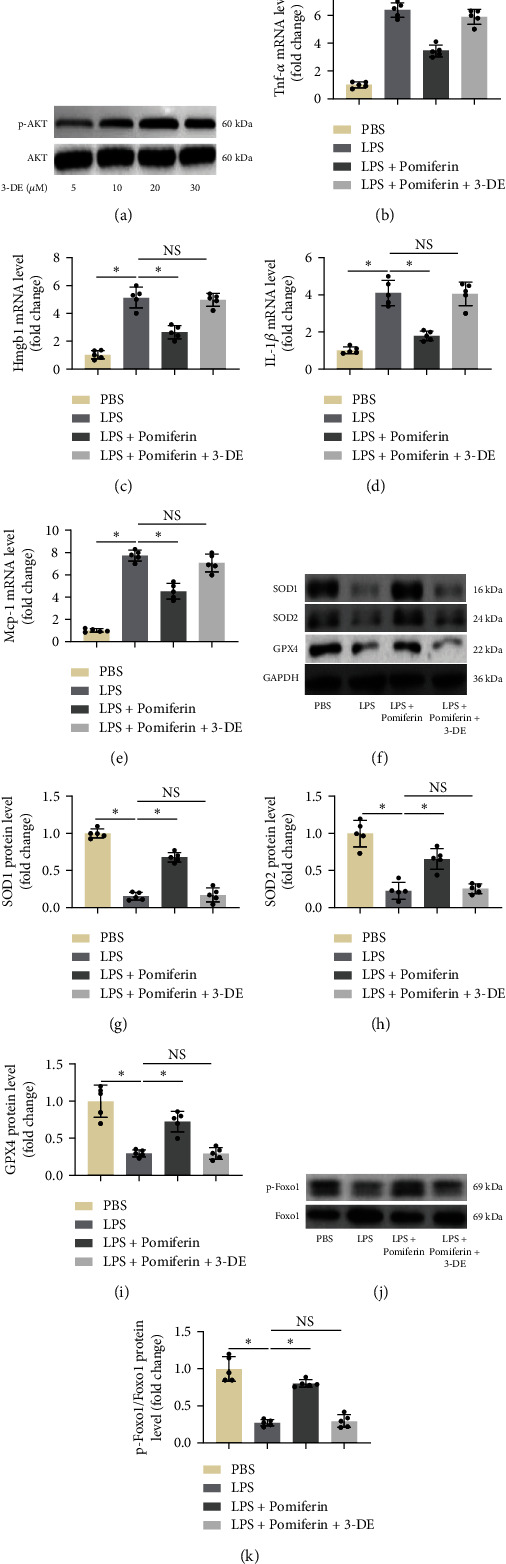
Pomiferin inhibited LPS-induced inflammation and oxidative stress in RAW264.7 macrophages in an AKT/Foxo1 pathway-dependent manner. (a) Representative western blots of proteins of p-AKT and AKT in 3-DE-treated RAW264.7 macrophages (*n* = 5). (b–e) The mRNA levels of *Tnf-α*, *Hmgb1*, *Mcp-1*, and *Il-1β* in RAW264.7 macrophages determined by RT-qPCR (*n* = 5). (f–i) Representative western blots and semiquantitative results of proteins of SOD1, SOD2, and GPX4 in RAW264.7 macrophages (*n* = 5). (j, k) Representative western blots and semiquantitative results of proteins of p-FOXO1 and FOXO1 in RAW264.7 macrophages (*n* = 5). ^∗^*P* < 0.05 vs. the indicated groups, NS means no significance.

To further verify the protection of pomiferin in LPS-induced inflammation and oxidative stress is mediated by AKT/Foxo1 pathway, we used different concentrations of 3-DE to activate AKT in RAW264.7 macrophages.  Figure 8(a) showed that 3-DE could significantly activate AKT in RAW264.7 macrophages. In particular, 30 *μ*M of 3-DE showed more potent effects than other concentrations. Next, we used 30 *μ*M of 3-DE to activate AKT in RAW264.7 macrophages. RT-qPCR showed that pomiferin treatment could significantly decrease the mRNA levels of proinflammatory genes including *Tnf-α*, *Hmgb1*, *IL-1β*, and *Mcp-1* in LPS-treated RAW264.7 macrophages, which could be completely offset after AKT activation (Figures 8(b)–8(e)). In addition, pomiferin also suppressed oxidative stress in LPS-treated RAW264.7 macrophages, evidenced by increased protein levels SOD1, SOD2, and GPX4 (Figures 8(f)–8(i)). Similarly, AKT activation abolished the effects of pomiferin completely. Finally, we detected the protein expression of p-Foxo1 and Foxo1. The result further showed that pomiferin enhanced the phosphorylation of Foxo1 in an AKT-dependent manner in LPS-treated RAW264.7 macrophages (Figures 8(j) and 8(k)).

## 4. Discussion

**Figure 9:**
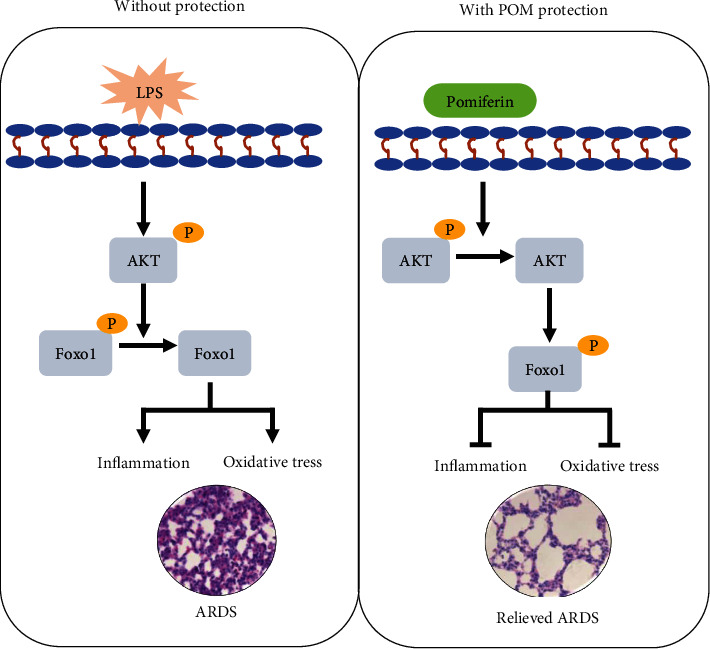


In the present study, we found that pomiferin treatment could alleviate the severity of acute respiratory distress syndrome by blocking inflammation and oxidative stress in an AKT-dependent manner (Figure 9). Our study disclosed pomiferin may serve as a potential therapy or adjuvant therapy for ARDS in the future.

ARDS is a life-threatening condition syndrome characterized by acute lung pathological injury and hypoxemia, with a high mortality of over 35% [33, 34]. A great many pathological conditions are associated with ARDS, including sepsis, pneumonia, trauma, shock, and ventilator-induced lung injury [34]. In terms of pathogenesis, inflammation, oxidative stress, and epithelial barrier impairment are three main mechanisms contributing to ARDS [35]. Therefore, drugs with the potentials of suppressing inflammation and oxidative stress and improving pulmonary function are promised to be therapeutic candidates against ARDS.

Macrophages in lung tissues play essential roles in inflammatory response and tissue injury during ARDS. Alveolar macrophages and interstitial macrophages are two main subpopulations of macrophages in lung tissues [36]. In terms of quantity, alveolar macrophages are more abundant than interstitial macrophages, which act as the first line of defense combating foreign invading factors. Hence, alveolar macrophages exert very important roles in the maintenance of immune homeostasis as well as host defense in the pulmonary local microenvironment. Upon stimulation by foreign pathogens, a variety of inflammatory chemokines could be released by alveolar macrophages, subsequently initiating a cascade of amplified inflammatory responses and mediating lung pathological injury as well as systemic inflammation [37, 38]. Interstitial macrophages are differentiated from alveolar macrophages by their localization, regulating immune reactions and participating in the inflammation process. During ARDS, both alveolar macrophages and interstitial macrophages are activated, which could then release excessive proinflammatory cytokines and result in lung tissue injury [39, 40]. ROS is a very important factor driving inflammatory response, which performs an integral role in the development of ARDS. Excessive free radicals could aggravate immune signals and intensify the tissue damage [41]. Superoxide dismutases (SODSs) serve as a group of metalloenzymes which could catalyze the dismutation of superoxide radicals into oxygen and hydrogen peroxide [42]. SODs have been reported to combat oxidative stress-related diseases by alleviating ROS-mediated damage [43]. During sepsis-induced ARDS, LPS stimulation significantly increased the ROS level in J774.A1 macrophages. However, ROS scavenger could relieve LPS-induced inflammatory response in J774.A1 macrophages, suggesting that oxidative stress could further aggravate inflammation in LPS-treated macrophages [44]. Here, our data showed that pomiferin could significantly decrease mRNA levels of proinflammatory cytokines including *Tnf-α*, *Hmgb1*, *Mcp-1*, and *Il-1β* in vivo and in vitro. Meanwhile, pomiferin also upregulated the protein levels of antioxidant enzymes including SOD1, SOD2, and GPX4 in lung tissues from mice with ARDS and LPS-treated macrophages. These results hinted that pomiferin exerted pulmonary protection by decreasing inflammation and oxidative stress during ARDS.

AKT is a critical protein kinase B regulating cell survival, inflammation, oxidative stress, and cell death, playing a crucial role in solid organ injury [16]. Phosphorylated Foxo1 could be dephosphorylated to form Foxo1 once AKT is activated, leading to its nuclear translocation [45]. It has been demonstrated that both the activation of the AKT and the reduction of Foxo1 expression in the nucleus can give rise to lung pathological injury and ARDS [18]. Here, we found that LPS stimulation significantly promoted the phosphorylation of AKT and the dephosphorylation of Foxo1 in macrophages and lung tissues from mice with ARDS. And pomiferin pretreatment could significantly inhibit the phosphorylation of AKT but promote the phosphorylation of Foxo1 in LPS-treated macrophages and lung tissues. In addition, AKT activation by 3-DE could completely offset the antioxidant and the anti-inflammatory effects of pomiferin in LPS-treated macrophages, suggesting that the protection of pomiferin during ARDS is mediated by AKT. However, there are some limitations in our study. Multiple cell types including macrophages, lung epithelial cells, and vascular endothelial cells participated in the development of ARDS. In our study, we only focused on the effects of pomiferin on macrophages. Whether pomiferin shows protective effects on lung epithelial cells and vascular endothelial cell needs further exploring. In addition, AKT is a relatively upstream molecule that could regulate a great many downstream signals. Whether AKT inhibition mediated by pomiferin could affect other signals also needs investigating in the future.

In conclusion, this study disclosed that pomiferin could alleviate the severity of ARDS in vitro and in vivo through regulating AKT/Foxo1 pathway in macrophages. Meanwhile, pomiferin also plays a critical role in suppressing LPS-induced pulmonary edema and lung pathological injury by decreasing inflammatory response and oxidative stress. Thus, pomiferin could be a promising and effective therapeutic candidate for the treatment of ARDS.

## Figures and Tables

**Table 1:** Primers used for RT-qPCR in this study.

Target gene	Forward primer	Reverse primer
Mus GAPDH	ACGTAGTGCTGCTCAAA	CATGCTGTAGTAGATCCCAA
Mus IL-1*β*	GCAACTGTTCCTGAACTCAACT	ATCTTTTGGGGTCCGTCAACT
Mus MCP-1	ACTGATCGTGATAGTAGCC	TCGTAGTGCTAGAAACCCA
Mus TNF-*α*	AAGCCTGTAGCCCACGTCGTA	GGCACCACTAGTTGGTTGTCTTTG

## Data Availability

The data that support the findings of this study are available from the corresponding author upon reasonable request.
